# Correlation of Color Fundus Photograph Grading with Risks of Early Age-related Macular Degeneration by using Automated OCT-derived Drusen Measurements

**DOI:** 10.1038/s41598-018-31109-x

**Published:** 2018-08-28

**Authors:** Chui Ming Gemmy Cheung, Yuan Shi, Yih Chung Tham, Charumathi Sabanayagam, Kumari Neelam, Jie Jin Wang, Paul Mitchell, Ching-Yu Cheng, Tien Yin Wong, Carol Yim Lui Cheung

**Affiliations:** 1 Singapore Eye Research Institute, Singapore National Eye Centre, Singapore, Singapore; 2 Ophthalmology & Visual Sciences Academic Clinical Program (Eye ACP), Duke-NUS Medical School, Singapore, Singapore; 3 Ophthalmology and Visual Sciences, Khoo Teck Puat Hospital, Singapore, Singapore; 4 Department of Ophthalmology, National University of Singapore, Singapore, Singapore; 5 Centre for Vision Research, Westmead Institute for Medical Research, University of Sydney, Sydney, New South Wales Australia; 6 Department of Ophthalmology, Chinese University of Hong Kong, Ma Liu Shui, Hong Kong

## Abstract

We evaluated automated OCT-derived drusen volume measures in a population-based study (n = 4,512) aged ≥40 years, and its correlation with conventional color fundus photographs (CFP)-derived early AMD features. Participants had protocol-based assessment to capture medical and ocular history, genotyping for SNPs in *CFH*, *ARMS2*, and *CETP*, CFP-based AMD grading and automated drusen volume based on SD-OCT using built-in software (Cirrus OCT advanced RPE analysis software). Significantly fewer eyes with early AMD features (drusen, hyperpigmentation, soft or reticular drusen) had drusen volume = 0 mm^3^ (p < 0.001). In eyes with drusen volume > 0 mm^3^, increasing AMD severity was associated with increase in drusen volume (correlation coefficient 0.17, p < 0.001). However 220 (59.14%) of 372 participants with AMD based on CFP grading had drusen volume = 0 mm^3^. Factors associated with drusen volume included age (OR 1.42 per 5 years, 95% confidence interval [CI] 2.76, 4.48), systolic blood pressure (OR1.00, 95% CI 1.00, 1.01), ethnic Malay (OR 1.54, 95% CI 1.29, 1.83) and Chinese (OR 1.66, 95% CI 1.37, 2.01) compared to Indian. The *ARMS2* rs10490924 T allele was associated with increased drusen volume in subjects with AMD (multivariable adjusted OR1.54, 95% CI 1.08, 2.19). Automated OCT-derived drusen volume is correlated with CFP-based AMD grading in many, but not all subjects. However the agreement is not good. These two modalities provide complementary information and should be incorporated into future studies.

## Introduction

Drusen are extracellular deposits accumulating within Bruch’s membrane or between the retinal pigment epithelium (RPE) and Bruch’s membrane on the apical side of the RPE, and are considered the hallmark of age-related macular degeneration (AMD). Increasing severity of baseline drusen characteristics and pigmentary changes have been shown to be important predictors for progression of AMD^1,2^. The gold standard method for qualitative and quantitative assessment of drusen has traditionally been based on assessment of high quality stereoscopic color fundus photographs (CFP), typically using modifications of the Wisconsin AMD grading system^3^. This method has been employed in many population-based studies around the world, including the Beaver Dam and Blue Mountains Eye Studies^4^. However, there are limitations of CFP-based grading, both due to technical reasons as well as due to the labor intensive, time-consuming nature of CFP assessment.

Optical coherence tomography (OCT), particularly spectral domain OCT (SD-OCT) is increasingly used in clinical studies and trials to evaluate AMD^5^. Recently, fully automated measurements of drusen volume from OCT have become possible with commercially available software. Several, mostly smaller clinic-based, studies have validated the accuracy and reproducibility of these automated OCT-based algorithms^5–9^. A few population-based studies have further correlated OCT-derived drusen measures with demographic risk factors^10^, genetic variants^11^ and risk of AMD progression^12^.

To date, there have been no studies correlating fully automated OCT-derived drusen volume measurements with conventional CFP grading in population studies in Asians. Previous clinical and epidemiological studies have suggested that drusen characteristics based on CFP grading in Asians and the risk they pose on AMD progression may be different from those in white populations^13,14^. In this study, we investigated the correlation of automated OCT-derived drusen volume with CFP-derived Wisconsin AMD grading and specific AMD features from a large population-based study. In addition, we evaluated associations of AMD risk factors with OCT-derived drusen volume.

## Methods

### Study Population

Data for this analysis were derived from the Singapore Epidemiology of Eye disease (SEED) program, a population-based cohort study of eye diseases in adults aged 40 years and older living in Singapore. Specifically, this report analyzes SD-OCT measurements, which were incorporated into the examination protocols since 2009. The SEED program was approved by the Institutional Review Board of the Singapore Eye Research Institute, Singapore and conducted in accordance with the Declaration of Helsinki. All participants provided written informed consent.

Detailed methodology and protocols for the examinations have been described elsewhere^15,16^. Briefly the sampling frame was composed of adults aged 40–80 years living in designated study areas in south-western Singapore during each stipulated study period, derived from a computer-generated list provided by the Singapore Ministry of Home Affairs using an age-stratified (by 10-year age groups) random sampling method.

### Ophthalmic examination and AMD grading

All participants had an interview, systemic examination, and laboratory investigations to determine socioeconomic, ocular, and systemic risk factors. After pupil dilation with 1.0% tropicamide, CFP were taken according to standardized protocol. Specifically, CFP of Early Treatment for Diabetic Retinopathy Study^17^ standard fundus fields 1 (centered on the optic disc) and 2 (centered on the fovea) were obtained for both eyes using a digital retinal camera (Canon CR-1 Mark-II Nonmydriatic Digital Retinal Camera, Canon). The Centre for Vision Research, University of Sydney, performed AMD grading using the Wisconsin Age-Related Maculopathy Grading System modified for magnification^3,18^.

AMD status was classified per eye: early AMD was defined as presence of either any soft drusen (distinct or indistinct) plus pigmentary abnormalities, reticular drusen, or large soft drusen >125 µm in diameter with drusen area >500 µm-diameter. Late AMD was defined as the presence of neovascular AMD or geographic atrophy (GA). Neovascular AMD included serous or hemorrhagic detachment of the RPE or sensory retina, and the presence of subretinal or sub-RPE hemorrhages or subretinal fibrous scar tissue^3,15,16,19,20^. GA was characterized by sharply edged, roughly round or oval areas of RPE hypopigmentation, with clearly visible choroidal vessels. The minimum diameter of GA was 175 um, or larger^3,15,16,19,20^. All CFP were graded initially in a masked manner by two graders and discrepancies were adjudicated by a senior retinal specialist (PM).

### Optical Coherence Tomography Acquisition

Each eye was scanned after pupil dilation with Cirrus OCT 4000 with 512 A-scans × 128 B-scans over a 6- × 6-mm^2^ area, centered on the fovea. RPE analysis was performed using Cirrus Version 6.0 Advanced RPE Analysis algorithm. Quantitative measurement of drusen volume was obtained for the retinal region centered on the fovea with diameter of 5 mm, derived from the distance between the real RPE and the interpolated RPE floor. Six hundred and sixty-one eyes were excluded because of low signal strength (<6) or presence of pathology which was thought could affect the accuracy of RPE-fit line which represents the logarithm-determined Bruch’s membrane (vitreomacular traction, epiretinal membranes or extensive peripapillary atrophy).

### Assessment of Risk Factors

A detailed interviewer-administered questionnaire was used to collect information about medical history (including hypertension, diabetes, angina, myocardial infarction and stroke), cigarette smoking (defined as current, past and never). The following information was collected: demographic information, socioeconomic characteristics (education, income level and occupation), family and medical history, and lifestyle factors (smoking, alcohol). We defined age as the age at examination. We categorized cigarette smoking into current smokers, former smokers or nonsmokers. Alcohol consumption was categorized into drinkers (those who reported having drunk alcohol in the past 3 months, irrespective of quantity) and non-drinkers. The participant’s height was measured in centimeters using a wall-mounted measuring tape. Weight was measured in kilograms using a digital scale (SECA, model 7822321009: Vogel & Halke, Hamburg, Germany). We calculated body mass index (BMI) using weight divided by the square of body height. Blood pressure was taken with the participant seated and after 5 minutes of rest. Systolic and diastolic blood pressure and pulse rate were measured with a digital automatic blood pressure monitor (Dinamap model Pro Series DP110X-RW, 100V2; GE Medical Systems Information Technologies Inc., Milwaukee, USA). Blood pressure was measured on two occasions 5 minutes apart. If the blood pressures differed by more than 10 mmHg in systolic or 5 mmHg in diastolic, a third measurement was taken. The blood pressure of the participant was defined as the mean between the two closest readings. A 40 mL sample of non-fasting venous blood was collected for the assessment of Hemoglobin A1c (HbA1c), serum glucose and lipid levels. All serum biochemistry tests were performed in the Singapore General Hospital Laboratory on the same day.

### Genotyping

We extracted DNA from venous blood samples from all participants. Genotyping was performed using Illumina Human OmniExpres or Human Hap610-Quad Beadchip. For the current analysis, we evaluated the following four SNPs: CFH rs900292, *ARMS2* rs10490924, *CETP* rs3764261 and rs2303790 (D442G). Genetic ancestry was inferred using principal component (PC) analysis to account for spurious associations resulting from ancestral differences of individual SNPs. Details of the PC analysis is reported in detail elsewhere^21,22^.

### Statistical Methods

All the statistical analysis was performed using R software version 3.2.2^23^. In order to account for the highly skewed distribution of drusen volume, the dataset was dichotomized into 2 categories: Drusen volume = 0 (n = 7,312) and Drusen volume > 0 mm^3^ (n = 1, 301) and the association between exposure variables (e.g. age, gender). Relationship between drusen volume and CFP-derived AMD outcomes (early and late AMD) and specific early AMD features was first assessed through trend test which tests the hypothesis of an increased trend of drusen volume with each ordered individual CFP grading parameter, using non-parametric Cuzik’s trend test^23,24^ for continuous response, and Cochran-Armitage test^25,26^ for binary response of drusen volume (cutoff at 0 mm^3^). As Cuzik’s trend test assumes no correlation in the dataset, only the worse eye is selected from each participant for the test (n = 3795 and n = 717 for Drusen volume = 0 and > 0 mm^3^, respectively). In addition, the strength of association between Drusen volume and AMD was also evaluated using Spearman rank correlation test. In regression analysis, drusen volume of both eyes (outcome variable) was assessed using logistic regression analysis with generalized estimating equations to account for the inter-correlation between left and right eyes. The association is firstly assessed with each single exposure factor, and then, the association was further controlled for the factors which are significant in the univariable model, including age, gender, ethnicities, LDL, anti-cholesterol medication, history of cardiovascular disease, creatinine, glomerular filtration rate, blood pressure and genetic factors, plus factors and AMD status. The association between genetic factors (including *CFH*, *ARMS2*, *CETP* and *D442G*) and drusen volume was assessed through logistic regressions in participants stratified by AMD status. Univariable and multivariable logistic regression models were constructed after no adjustment or adjusting for age, gender and principal components 1–2 from principal component analysis of genetic data.

## Results

We included 4,512 subjects of Chinese (n = 1, 294) and Malay (n = 1, 346) and Indian (n = 1, 872) ethnicity. The mean age was 59.1 years. Of the 4,521 participants, 348 had early AMD and 24 subjects had late AMD in the worse eye. Bilateral AMD was present in 92 participants. For *CFH*, 486 subjects (14.79%) were homozygous and 1, 552 subjects (47.25%) were heterozygous for rs800292_A respectively. For *ARMS2*, 479 subjects (14.69%) were homozygous and 1, 562 (47.91%) were heterozygous for the rs104909274_T. For *CETP*, 151 subjects (4.60%) were homozygous and 1, 027 subjects (31.24%) were heterozygous for rs3764261_A; whereas 1 subject (0.03%) was homozygous and 54 subjects (1.67%) were heterozygous for D442G. Detailed baseline characteristics are summarized in Table 1.

**Table 1:** Characteristics of study participants.

	Mean/Number (SD/%)
AGE, years	59.1 (8.6)
GENDER, Female	2307 (51.1%)
Ethnicity	
Chinese	1294 (28.7%)
Malay	1346 (29.8%)
Indian	1872 (41.5%)
Body Mass Index, kg/m2	25.79 (4.66)
Total cholesterol, mmol/L	5.36 (1.18)
Blood HDL Cholesterol	1.26 (0.34)
Blood LDL Cholesterol	3.43 (0.98)
Blood suger level(SD)	6.90 (3.22)
Creatinine,	76.77 (42.42)
HbA1c, %	6.25 (1.28)
Systolic Blood pressure, mmHg	135.59 (18.52)
Diastolic Blood pressure, mmHg	77.73 (9.86)
Smoking status	
Never smoked	3151 (72.7%)
Current smoker	656 (15.1%)
Past smoker	525 (12.1%)
Drinking alcohol, yes	384 (8.9%)
History of cardiovascular disease, yes	393 (9.1%)
Use anti-cholesterol medication, yes	1461 (34.4%)
**Genetic risk factors**	
*CFH* rs900292 (*n = 3,285)	
CC	1247 (37.96%)
AC	1552 (47.25%)
AA	486 (14.79%)
*ARMS2* rs10490924 (*n = 3,260)	
GG	1221 (37.45%)
GT	1562 (47.91%)
TT	479 (14.69%)
*CETP* rs3764261 (*n = 3,287)	
CC	2109 (64.16%)
AC	1027 (31.24%)
AA	151 (4.60%)
*D442G* rs2303790 (*n = 3,286)	
TT	3231 (98.33%)
GT	54 (1.64%)
TT	1 (0.03%)

*Total number of subjects with genotype data available for each SNP studied.

Drusen volume was markedly skewed towards 0 mm^3^. Drusen volume was 0 mm^3^ in 3,795 (84.11%) of 4,512 participants, and 3,575 (94.20%) had no AMD according to CFP grading. In the remaining 717 participants with drusen volume > 0 mm^3^, only 152 (21.20%) had AMD based on CFP grading. Despite this skewed deviation, the proportion of eyes with drusen volume = 0 mm^3^ significantly decreased from no AMD (86.36%) to early AMD (62.25%) to late AMD (12.5%), p < 0.001 The correlation coefficient between drusen volume and AMD status in all 4,512 participants is 0.21 (p < 0.001). In participants in whom early AMD features evaluated were present (any drusen, pigmentation, soft and reticular drusen), there were also significantly lower percentage of participants with drusen volume = 0 mm^3^ than that in participants with no AMD feature (p < 0.001). Similarly, with increasing drusen size, number and area, significantly fewer proportion of participants have drusen volume = 0 mm^3^ p < 0.001. Among the remaining 717 participants with drusen volume > 0 mm^3^, there was a significant trend of increasing drusen volume as AMD severity increases (correlation coefficient 0.17, p < 0.001). Among the individual early AMD features evaluated, drusen volume was significantly increased in eyes with reticular drusen, p = 0.005. (Table 2)

**Table 2:** OCT-derived Drusen volume and color fundus photograph-derived AMD category and individual AMD Features.

	Total N = 4512	Number (%) of eyes with drusen volume = 0 mm^3^	P-trend^*^ of eyes with dichotamized drusen volume	Drusen Volume			P-trend^Ϯ^ of eyes with DV > 0 mm^3^	P-trend^Ϯ^ (Overall)
				(In 717 participants with DV > 0 mm^3^)				
				25% Percentile	Median	75% Percentile		
AMD status								
No AMD	4141	3576 (86.36%)	**<0.001**	0.002	0.007	0.024	**0.036**	**<0.001**
Early AMD	347	216 (62.25%)		0.002	0.006	0.019		
Late AMD	24	3 (12.50%)		0.004	0.121	0.459		
Any Drusen								
None	3924	3388 (86.34%)	**<0.001**	0.002	0.008	0.02725	0.994	**<0.001**
Yes	588	407 (69.22%)		0.002	0.005	0.018		
Hyperpigmentation								
None	3948	3427 (86.80%)	**<0.001**	0.002	0.007	0.026	0.908	**<0.001**
Yes	564	368 (65.25%)		0.002	0.006	0.022		
Soft drusen								
None	3147	2804 (89.10%)	**<0.001**	0.002	0.007	0.023	0.492	**<0.001**
Intermediate	459	337 (73.42%)		0.001	0.005	0.018		
Soft distinct	129	71 (55.04%)		0.002	0.006	0.022		
Soft Indistinct	631	502 (79.56%)		0.002	0.006	0.026		
Reticular drusen								
None	4439	3760 (84.70%)	**<0.001**	0.002	0.007	0.023	**0.005**	**<0.001**
Yes	9	1 (11.11%)		0.017	0.022	0.118		
Max Drusen size								
None	927	791 (85.33%)	**<0.001**	0.002	0.007	0.028	0.909	**<0.001**
<63 µm	2190	1993 (91.00%)		0.002	0.007	0.021		
≥63-<125 µm	646	515 (79.72%)		0.002	0.006	0.025		
≥125-<250 µm	542	392 (72.32%)		0.001	0.004	0.017		
≥250 µm	43	13 (30.23%)		0.004	0.015	0.071		
Drusen number								
None	926	790 (85.31%)	**<0.001**	0.002	0.007	0.028	0.888	**0.002**
<10	2411	2128 (88.26%)		0.002	0.007	0.022		
≥10	1010	785 (77.72%)		0.002	0.006	0.022		
Total drusen area								
<63 µm	939	797 (84.88%)	**<0.001**	0.002	0.007	0.028	0.721	**<0.001**
63–125 µm	1876	1701 (90.67%)		0.002	0.007	0.021		
125–175 µm	894	769 (86.02%)		0.003	0.007	0.024		
175–350 µm	276	201 (72.83%)		0.001	0.004	0.018		
350–650 µm	241	171 (70.95%)		0.001	0.004	0.01		
<Half DA	65	38 (58.46%)		0.003	0.008	0.019		
≥Half and <1DA	23	15 (65.22%)		0.002	0.004	0.019		
≥1 DA	41	15 (36.59%)		0.005	0.018	0.073		

Ptrend* of eyes with DV = 0 VS DV > 0 mm^3^: after dichotamizing drusen volumes at 0, test for increasing trend of drusen volume with increased exposure was conducted for binary response using Cochran-Armitage test.

Ptrend* of eyes with DV > 0 mm^3^ or total dataset: test for increasing trend of drusen volume with increased exposure was conducted for continuous response using Cuzik’s trend test.

P values < 0.05 are highlighted by bold font.

Next, the association of drusen volume with possible AMD risk factors was assessed using logistic regression. In the univariate regression model, drusen volume was associated with age, total cholesterol, LDL-cholesterol, anti- hyperlipidemia medication, glomerular filtration rate, systolic blood pressure, history of cardiovascular disease, and ethnic Malay (compared to Indian ethnicity) and presence of any AMD. (Table 3) In the multivariable regression model, higher drusen volume was associated with older age (OR 1.42 per 5 years, 95% CI 2.76, 4.48), systolic blood pressure (OR 1.00, 95% CI 1.00, 1.01), ethnic Malay (OR 1.54, 95% CI 1.29, 1.83) and Chinese (OR 1.66, 95% CI 1.37, 2.01) compared to Indian and presence of any AMD (OR 3.52, 95% CI 2.76, 4.48) remained significantly associated with higher drusen volume.

**Table 3:** Factors influencing OCT-derived drusen volume.

	Univariable-model		Multivariable-model ^*^	
	OR (95% CI)	P-val	OR (95% CI)	P-val
Any AMD, yes	4.05 (3.27 5.02)	**<0.001**	3.52 (2.76 4.48)	**<0.001**
Age, every 5 years	1.48 (1.42 1.54)	**<0.001**	1.42 (1.35 1.49)	**<0.001**
Gender, reference to Male	1.00 (0.88 1.15)	0.954	1.14 (0.98 1.32)	0.101
Body mass index, kg/m^2^	1.00 (0.98 1.01)	0.558	—	—
Total cholesterol, mmol/l	0.89 (0.84 0.95)	**<0.001**	—	—
HDL cholesterol, mmol/l	1.10 (0.9 1.34)	0.352	—	—
LDL cholesterol, mmol/l	0.83 (0.77 0.9)	**<0.001**	0.97 (0.89 1.05)	0.412
Anti-hyperlipidaemia medication	1.47 (1.28 1.7)	**<0.001**	1.01 (0.84 1.21)	0.922
Blood glucose, mmol/l	1.02 (1.00 1.04)	0.073	—	—
Serum Creatinine, mmol/l	1.00 (1.00 1.00)	0.001	1 (1 1)	0.979
Glomerular Filtration Rate,	0.98 (0.98 0.98)	**<0.001**	1.00 (0.99 1.00)	0.546
HbA1c, %	1.05 (1.00 1.11)	0.056	—	—
Axial length, mm	1.02 (0.94 1.1)	0.641	—	—
Systolic blood pressure, mmHg	1.01 (1.01 1.02)	**<0.001**	1.00 (1.00 1.01)	**0.036**
Diastolic blood pressure, mmHg	0.99 (0.99 1.00)	0.046	—	—
Alcohol consumption, yes	0.84 (0.66 1.08)	0.17	—	—
History of cardiovascular disease, yes	1.40 (1.14 1.74)	**0.002**	0.94 (0.74 1.21)	0.64
Non-smoker	Reference	Reference	Reference	Reference
Past smoker	0.92 (0.76 1.11)	0.385	—	—
Current smoker	1.14 (0.93 1.40)	0.194	—	—
Indian	Reference	Reference	Reference	Reference
Malay	1.49 (1.27 1.74)	**<0.001**	1.54 (1.29 1.83)	**<0.001**
Chinese	0.98 (0.82 1.16)	0.794	1.66 (1.37 2.01)	**<0.001**

The analysis was done using logistic model for binary outcome of drusen volume based on: Drusen volume = 0 (n = 7372); Drusen volume > 0 (n = 1301).

*Multivariable models adjusted for age, gender, ethnicities, LDL, anti-cholesterol medication, history of cardio vascular disease, Glomerular Filtration Rate, blood pressure, and AMD.

P values < 0.05 are highlighted by bold font.

Amongst the AMD risk genotypes evaluated, presence of the *ARMS2* rs10490924 T allele was associated with increased drusen volume (multivariable adjusted OR1.54, 95% CI 1.08, 2.19) in subjects with AMD (n = 347). (Table 4) None of the genotypes evaluated was significantly associated with increased drusen volume in subjects without AMD.

**Table 4:** Genotype association with optical coherence tomography (OCT)-derived drusen volume in participants with AMD (n = 347).

	Number (%) with Drusen volume = 0	Number (%) with Drusen volume > 0	Model 1*		Model 2*	
			*OR (95% CI)*	*P_val*	*OR (95% CI)*	*P_val*
***CFH*** **rs900292**			0.82 (0.58 1.16)	0.251	0.78 (0.53 1.14)	0.203
CC	71(54.62%)	59(45.38%)				
AC	104(61.9%)	64(38.1%)				
AA	29(59.18%)	20(40.82%)				
***ARMS2*** **rs10490924**			1.59 (1.14 2.24)	**0.007**	1.54 (1.08 2.19)	**0.017**
GG	83(68.6%)	38(31.4%)				
GT	96(58.18%)	69(41.82%)				
TT	25(40.98%)	36(59.02%)				
***CETP*** **rs3764261**			0.97 (0.66 1.42)	0.864	0.97 (0.65 1.45)	0.885
CC	130(59.09%)	90(40.91%)				
AC	62(56.36%)	48(43.64%)				
AA	12(70.59%)	5(29.41%)				
***D442G*** **rs2303790**			NA	NA	NA	NA
TT	203(58.67%)	143(41.33%)				
GT	1(100%)	0(0%)				
GG	0	0				

AMD Age-related macular degeneration; DV drusen volume; OR Odds ratio; CI confidence intervals.

*Multivariable ordinal regression model adjusted for age, gender and principal components 1–3 from principal component analysis of genetic data.

## Discussion

**Figure 1:**
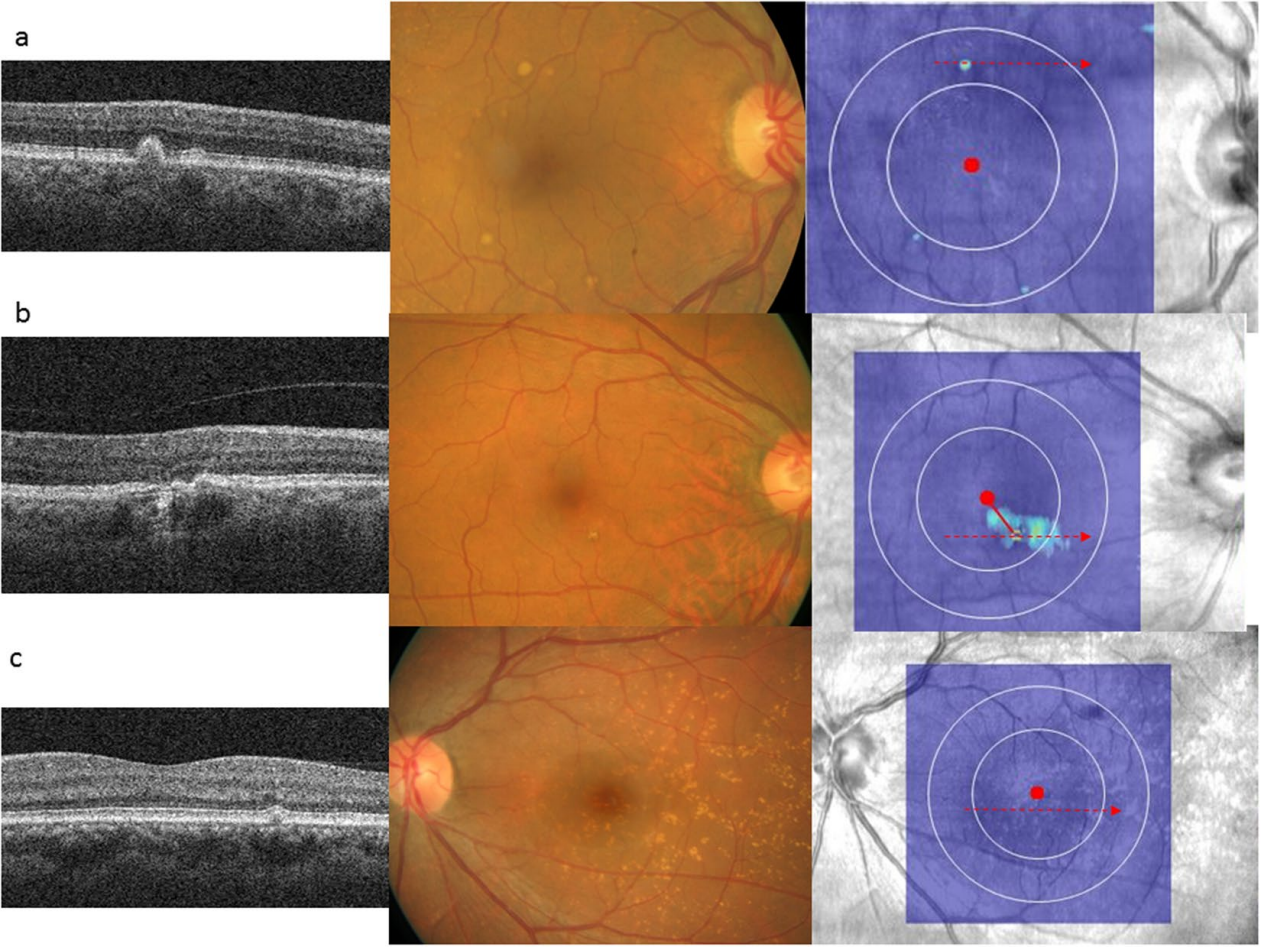
Examples of comparison between cross-sectional OCT through red dashed arrow, color fundus photographs (CFP) and OCT-derived drusen volume map. In (**a**), soft drusen can be seen on CFP and RPE elevation can be seen in corresponding cross-sectional OCT. This area was reflected as increased drusen volume (in turquoise) on the enface map. In (**b**), only hyperpigmentation can be seen on CFP. The corresponding cross-sectional OCT showed shallow and irregularly elevated RPE. A wider area of elevated drusen volume is highlighted in turquoise in the enface drusen volume map. In (**c**), cuticular (hard) drusen are seen on CFP, and very mild elevation of RPE is seen in a localized area. No significant elevation of drusen volume was reflect on the enface map.

In this population-based study using data from CFP and SD-OCT-derived drusen volume measured from 4512 subjects, we showed automated measurement of drusen volume increased with AMD severity based on traditional Wisconsin AMD grading parameters using CFP. We note that drusen volume was markedly skewed towards 0 mm^3^, and not surprisingly, majority of participants with no AMD based on CFP grading had drusen volume = 0 mm^3^. Interestingly, 220 (59.14%) of 372 participants with AMD based on CFP grading also had drusen volume = 0 mm^3^. (Fig. 1) This discrepancy is likely due to eyes with small drusen and shallow RPE distortion^27,28^. Conversely, 152 participants had drusen volume > 0 mm^3^ but no AMD based on CFP grading. Several small studies have previously compared drusen area and volume measured based on SD-OCT versus CFP and reported different levels of agreement between these two modalities. Jain *et al*. evaluated drusen area reported mean agreement in 82% in 12 eyes with non-neovascular AMD^27^. Freeman *et al*. also reported ‘strong and significant correlation’ between drusen volume and AREDS-determined drusen area in 36 eyes with non-exudative AMD, but added that the correlation is not perfect^28^. In contrast, Yehoshua *et al*. reported only fair agreement between drusen area measurements obtained from SD-OCT images and CFP based on 50 eyes with drusen secondary to non-exudative AMD^29^. Some disagreement between the two modalities could be explained by the different properties of drusen being detected, namely pigmentary changes on CFP as opposed to distortion in RPE geometry on SD-OCT. As a result, CFP may be less accurate, particularly in eyes with large drusen areas whereas SD-OCT may be less sensitive in eyes with small drusen and shallow RPE distortion^28,29^. The findings from the current study with the largest sample size comparing these two modalities further highlight that these two imaging modalities provide complementary information regarding RPE changes in AMD.

For several decades CFP-based grading has been the most widely-used imaging modality to assess AMD in population and clinical studies^3,19,20,30–33^. Features assessed include drusen characteristics as well as pigmentary changes for early AMD, whereas signs of pigment epithelial detachment, choroidal neovascularization and GA are the key features assessed for late AMD. AREDS 1 and AREDS2 reported that baseline drusen characteristics (including drusen area and maximum drusen size) and pigmentary abnormalities are strongly predictive of subsequent risk of development of neovascular AMD^2,34^. We recently also validated the predictive value of the AREDS simplified severity scale in the six-year incidence of late AMD in Singaporean Malays^20^ and in the 5- and 10-year incidence of AMD in the Blue Mountains Eye Study^35^. However, reliable CFP quantification may be challenging due to difficulty in identifying clear drusen borders, particularly in photographs with low contrast. Assessment of maximum drusen size based on estimation by visual comparison to a set of standardized circles lacks precision. Furthermore, categorical grading is somewhat arbitrary. Finally, detailed grading of these parameters by trained professional graders, usually in centralized reading centers (e.g. University of Wisconsin or University of Sydney) remains time-consuming, labor intensive and costly. With the advent of SD-OCT, detailed cross-sectional imaging of drusen and the RPE can be obtained, thus enabling a three-dimensional rather than two-dimensional assessment of the macula, which may improve the precision of AMD feature grading^20^. Several groups have evaluated manual and subsequently customized semi-automated and automated quantification strategies and reported satisfactory repeatability and reproducibility, particularly for drusen volume^5–9,36^. More recently, fully automated drusen-volume quantification software has become commercially available (Carl Zeiss Meditec Inc, Dublin, CA, USA). This software allows for much higher throughput quantitative assessments of drusen volume, with highly reproducible results^29,37^. Incorporating such automated drusen volume measurement in future population study protocols is likely a natural trend. The current study is the largest population study to-date to provide information on the agreement between CFP and automated drusen volume, and also provides information on other factors which may influence drusen volume.

Regarding factors which may influence drusen volume, the strongest association was seen with the presence of any AMD (OR 3.52, p < 0.001). However, we also observed that increasing drusen volume was associated with older age, systolic blood pressure, ethnic Malay and Chinese compared to Indian ethnicity. These results are in keeping with previous report by Diniz *et al*. in non-AMD subjects aged 50 + years in which perifoveal drusen area was associated with increasing age, systolic blood pressure and years of smoking^38^. Ethnic variations in AMD lesions have also been reported in previous studies based on CFP^14^. In the current study, we report that Chinese and Malay ethnicity were associated with higher drusen volume compared to Indian ethnicity. Thus further studies are warranted to further evaluate differences between ethnic groups.

The pathogenesis of AMD is complex and multi-factorial. One of the key mechanisms involves thickening of the RPE-BM complex and deposition of extracellular materials above or below the RPE, which is thought to lead to reduced transport of nutrients and lowered oxygen tension. We hypothesize that increased drusen volume may be observed in eyes affected by this process, which may in turn be related to an individual’s genetic background. The association between OCT-derived drusen measurements with SNPs in eight AMD-associated genes (*SYN3*, *LIPC*, *ARMS2*, *C3*, *CFB*, *CETP*, CFI and *CFH*) was evaluated in 216 subjects (432 eyes) with intermediate AMD from the Amish population^11^. In that study, a significant association of the *CFH* risk allele GG for SNP rs120238333 with drusen area as well as drusen volume and area of RPE atrophy was reported, but none of the other 7 AMD-associated gene SNPS tested were associated with any of the drusen measurements. In the current study, we evaluated the association between drusen volume and four AMD-related SNPS. We did not find an association between *CFH* rs900292 and drusen volume. However in subjects with AMD, we found that *ARMS2* rs10490924_T was associated with increased drusen volume. Studies with larger sample size will be needed to further evaluate the potential association between *ARMS2* rs104900292 and drusen volume.

Strengths of the current study include its population-based sample, the adoption of standardized Wisconsin AMD grading methodology for CFP identical to those used in the landmark Beaver Dam and Blue Mountains Eye studies. The use of standardized questionnaires to capture baseline demographic data allowed us to evaluate the influence of a comprehensive list of medical and social risk factors on drusen volume. The availability of genotype data in a large sample size provided a unique opportunity to evaluate the influence of AMD-associated genetic polymorphisms on drusen volume. There are limitations, however, that need to be discussed. The automated drusen volume measurement algorithm used in this study is based on automatic SD-OCT segmentation of the outer border of the RPE layer and compared with the interpolated RPE floor which correspond to a virtual RPE layer in contact with Bruch’s membrane and free of deformation. Segmentation error may occur in cases of mild RPE distortion or low RPE reflectivity. As the minimum cutoff for detection of RPE elevation is 19.5μm, small drusen could be missed. Differences are likely to exist between several custom semi-automated and automated algorithms for drusen measurement. However, the use of a commercially available, FDA-approved automated drusen quantification tool in SD-OCT makes the current approach attractive and broadly applicable in clinical practice. Finally, to account for the highly right-skewed drusen volume distribution, we analyzed the relationship between drusen volume and CFP grading using non-parametric Cuzik’s trend test^23,24^.

In conclusion, using population-based data with >8,000 eyes, we showed automated SD-OCT measurement of drusen volume using a commercial algorithm (Cirrus OCT; Carl Zeiss Meditec, Inc.) correlated closely with conventional CFP-derived AMD severity and individual AMD features. These two imaging modalities offer unique but complementary information and should be incorporated in future population and clinical studies. Our results suggest automated OCT-derived drusen volume can potentially be used in large scale population studies and big data analyses, such as UK Biobank, to screen for early AMD.
